# High Expression of Heat Shock Protein 90 Is Associated with Tumor Aggressiveness and Poor Prognosis in Patients with Advanced Gastric Cancer

**DOI:** 10.1371/journal.pone.0062876

**Published:** 2013-04-26

**Authors:** Jiahong Wang, Shuzhong Cui, Xiangliang Zhang, Yinbing Wu, Hongsheng Tang

**Affiliations:** Department of Abdominal Surgery, Affiliated Oncologic Hospital of Guangzhou Medical College, Guangzhou, Guangdong, China; Peking Union Medical College Hospital, Peking Union Medical College, Chinese Academy of Medical Sciences, China

## Abstract

The heat shock protein 90 (HSP90) is overexpressed and highly associated with poor prognosis in many malignancies. However, the role of HSP90 in gastric cancer has not been thoroughly elucidated. The aim of this study is to investigate the relationship of HSP90 expression with clinicopathological parameters and prognosis in advanced gastric cancer, and estimate the alteration of HSP90 expression after neoadjuvant chemotherapy. HSP90 and matrix metallopeptidase 9 (MMP-9) antigen expression was evaluated by immunohistochemistry in 322 advanced gastric carcinoma samples. The relationships between HSP90 and clinicopathological parameters and prognosis were analyzed. The response of HSP90 level was assessed in chemotherapeutic effect in 54 patients received 1–2 cycles of neoadjuvant chemotherapy. The positive expression of HSP90 was found to be 69.6% in 322 advanced gastric carcinoma samples. HSP90 protein expression was significantly associated with depth invasion (*P*<0.001), lymph node metastasis (*P*<0.001) and stage of disease (*P*<0.001). The positive rates of HSP90 expression were higher in both prominent serosal invasion group (*P*<0.001) and lymph node metastasis group (*P*<0.001). Moreover, HSP90 was significantly correlated with MMP-9 among 322 gastric cancer tissues (*P*<0.001). In univariate and multivariate analyses, HSP90 was an independent prognostic factor for both recurrence-free survival (RFS) and overall survival (OS). These results suggested that HSP90 may play an important role on tumor invasion, metastasis and prognosis, and might act as a promising target for prognostic prediction.

## Introduction

Gastric cancer is the fourth most commonly diagnosed cancer, and the second leading cause of cancer-related death worldwide [1]–[2]. Complete resection of the tumor and adjacent lymph nodes is the only effective curative treatment [3]. However, after a complete resection, the 5-year survival rate remains low, reported to be only 23% [4]. In addition, even the addition of novel, molecularly targeted therapies to standard chemotherapy provide only a limited survival benefit [5]. Therefore, identification of novel prognostic and predictive markers is important, in order to facilitate the selection of patients who are most likely to benefit from multimodal therapeutic approaches.

Heat shock proteins (HSPs) are a set of evolutionarily conserved proteins. Mammalian HSPs have been classified mainly in four families according to their molecular weight: HSP90, HSP70, HSP60 and small HSPs (15–30 kDa) that include HSP27. It has been known that HSPs are expressed in response to an array of stresses including hyperthermia, oxygen-derived free radicals, amino acid analogues, ethanol and heavy metals [6]. Recently, it has been showed that HSPs were significantly associated with cancers [7].

The heat shock protein (HSP) 90 is an important molecular chaperone for protein folding, intracellular disposition and proteolytic turnover of key regulators of cell growth and survival [8]. Many studies have demonstrated that HSP90 is overexpression in solid tumors, and has significant association with prognosis [9]–[11]. It has also reported that HSP90 expression is high in gastric caner cell lines [12], yet no large studies have been conducted on expression in human tumors and the association with clinicopathological variables. Therefore, the aim of our study is to demonstrate the relationship of HSP90 expression with clinicopathological parameters and prognosis in advanced gastric cancer, and examine the response of HSP90 expression to neoadjuvant chemotherapy.

## Materials and Methods

### Patients and Clinical Data

The study was approved by the Institutional Review Board and Human Ethics Committee of Affiliated Oncologic Hospital of Guangzhou Medical College. Written consent for using the samples for research purposes was obtained from all patients prior to surgery.

We analyzed tissue from 322 patients (197 males and 125 females) with advanced gastric carcinoma who were treated at the Affiliated Oncologic Hospital of Guangzhou Medical College (Guangzhou, China) between 2005 and 2011. The age distribution was from 22 to 84 years, and the mean age was 56.11±11.42 years. According to the anaplastic grade, 15 tumors were well differentiated, 102 were moderately differentiated, and 205 were poorly differentiated. All the advanced gastric cancer patients enrolled in the study had a histologically confirmed diagnosis that the tumor has invaded gastric muscular or serosal layer, which was pathologically confirmed after surgery. Relevant clinical pathologic features (Table 1) were all obtained from the patients’ files. Tumor stage was classified according to the 7th Union International Cancer Control (UICC) TNM staging system [13]. The cases were selected consecutively on the basis of availability of resection tissues and follow-up data. One hundred and fifty-seven in 322 patients who were followed for at least 36 months had complete follow-up data, which were used for evaluating the prognosis. Furthermore, 54 cases in 322 patients underwent neoadjuvant chemotherapy, while the others did not receive radio- or chemotherapy prior to gastrectomy. These 54 patients all submitted to a diagnostic biopsy before neoadjuvant chemotherapy, and matched tumor specimens were obtained during surgical resection. All the specimens from 54 patients had enough residual tumor tissues to evaluate. A total of 322 advanced gastric carcinoma samples were used in the immunohistochemistry (IHC) analysis.

**Table 1:** Clinicopathologic Correlation of HSP90 Expression in 322 Gastric Cancer.

		HSP90 expression (%)		
Characteristics	Number	Low	High	*P*-value
Gender				
Male	197	58 (29.4%)	139 (70.6%)	
Female	125	40 (32.0%)	85 (68.0%)	0.627
Age (years)				
≤60	213	72 (33.8%)	141 (66.2%)	
>60	109	26 (23.9%)	83 (76.1%)	0.066
Size (cm)				
≤5.0	219	80 (36.5%)	139 (63.5%)	
>5.0	103	18 (17.5%)	85 (82.5%)	**0.001**
Tumor site				
Upper	72	9 (12.5%)	63 (87.5%)	
Middle/Lower	250	89 (35.6%)	161 (64.1%)	**<0.001**
Differentiation				
Well/Moderate	117	32 (27.4%)	85 (72.6%)	
Poor	205	66 (32.2%)	139 (67.8%)	0.364
Depth of invasion				
T2	144	80 (55.6%)	64 (44.4%)	
T3/T4	178	18 (10.1%)	160 (89.9%)	**<0.001**
Lymph nodemetastasis				
Negative	100	81 (81.0%)	19 (19.0%)	
Positive	222	17 (7.7% )	205 (92.3%)	**<0.001**
Stages				
I/II	125	78 (62.4%)	47 (37.6%)	
III/IV	197	20 (10.2%)	177 (89.8%)	**<0.001**

### Preoperative Chemotherapy

The initial diagnosis of 54 patients was done after January 2010. Patients with stage III-IV (M0) gastric cancer were enrolled for neoadjuvant chemotherapy. Pretreatment tumor staging was performed according to a uniform protocol that included endoscopy with endosonography, a CT scan of the chest and abdomen, and diagnostic laparoscopy.

All 54 patients were treated with the FOLFOX regimen (leucovorin, 5-fluorouracil, and oxaliplatin) [14] and underwent gastrectomy with D2 lymphadenectomy after chemotherapy. Neoadjuvant chemotherapy consisted of intravenous infusion of oxaliplatin (85 mg/m^2^, 2 h) and leucovorin (200 mg/m^2^, 2 h), followed by 5-fluorouracil 2600 mg/m^2^ as a 24 h continuous infusion. Cycles were repeated at 2-week intervals for one or two cycles. Surgical tumor resection was scheduled 2 weeks after the completion of chemotherapy. There were 16 patients who received one cycle of FOLFOX regimen, while others received two cycles of neoadjuvant chemotherapy.

### IHC Staining

We used a previously described IHC procedure [15]. Briefly, formalin-fixed, paraffin-embedded tissue samples from consenting patients were cut in 4-µm sections and placed on polylysine-coated slides, deparaffinized in xylene, and rehydrated in a series of graded alcohols. The tissue slides were then treated with 3% hydrogen peroxide in methanol for 10 min to quench endogenous peroxidase activity, and the antigens were retrieved in 0.01 M sodium citrate buffer (pH 6.0) using a microwave oven. After 30 min of preincubation in 10% normal goat serum to prevent nonspecific staining, the samples were incubated overnight using a primary antibody, either anti-HSP90 (Abcam, #ab13495, UK, dilution 1∶200) or anti-MMP-9 (Abcam, #ab38898, UK, dilution 1∶200), in a humidified container at 4°C. The tissue slides were treated with a non-biotin horseradish-peroxidase detection system according to the manufacturer’s instructions (Gene Tech). The IHC results were evaluated by two independent investigators blinded to the patients’ identity and clinical status. In discrepant cases, a pathologist reviewed the cases, and a consensus was reached.

**Figure 1:**
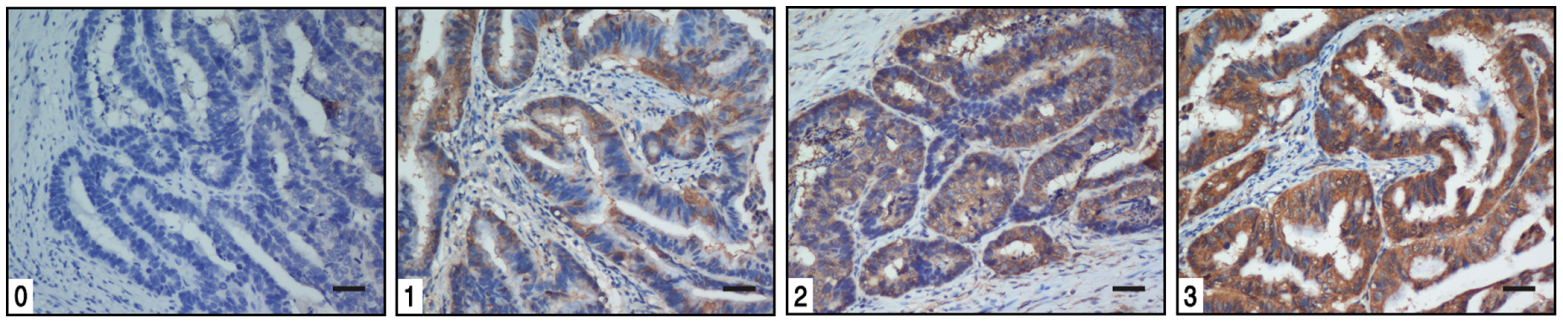
Gastric cancer tissue illustrating the range of intensities of HSP90 immunostaining from 0 to 3. The scale bar represents 100 µm.

HSP90 and MMP-9 staining intensities were rated on a scale of 0–3 according to the percentage of positive tumor (0, <5% positive cells; 1, 5–20%; 2, 20–50%; or 3, >50%). The expression is very low for 0, low for 1, moderate for 2 and high for 3 (Fig. 1). HSP90 and MMP-9 expression were classified as negative for scores ≤1 and positive for scores ≥2.

### Follow-up

Patients had follow-up appointments every 3 months for the first 3 years after surgery, every 6 months for the next 2 years, and yearly thereafter. The median follow-up period was 27 months (range, 4–82 months) in 322 patients, while there was 33 months in 157 patients whose prognosis was analyzed. Recurrence were confirmed by tumor markers levels including CEA, AFP, CA199, CA125 and CA724, B-type ultrasonic inspection every 3 moths, and computed tomography (CT) or magnetic resonance imaging (MRI) every 6 months after gastrectomy. The main causes of death were gastric cancer recurrence. Overall survival (OS) was calculated from the date of surgery to the date of death or last follow-up. Recurrence-free survival (RFS) was defined as from the date of surgery until the date of relapse or from the period of resection to the date of the last observation taken.

### Statistical Analysis

All statistical analyses were carried out using SPSS software (version 16.0; Chicago, IL, USA). Interdependence between HSP90 expression and clinical data was calculated using the chi-square test, and displayed in cross-tables. Group differences were examined using the Student’s *t*-test. Survival curves were plotted using the Kaplan-Meier method and analyzed using the log-rank test. Significant prognostic factors found by univariate analysis were entered into a multivariate analysis using the Cox proportional hazards model. Multiple comparisons were corrected by using the Bonferroni method. Two-tailed *P* values were calculated. Differences were considered statistically significant when *P*<0.05.

## Results

### The Association of HSP90 with Clinicopathological Variables

There were 322 cases of advanced gastric cancer who were investigated by immunohistochemistry. HSP90 staining mainly located in cytoplasm of tumor cells. Overexpression of HSP90 was observed in 224 of 322 (69.6%) of gastric cancer samples.

According to the results of immunohistochemistry, we correlated HSP90 status in 322 gastric cancer specimens with eight other widely recognized clinicopathologic parameters (Table 1). Our analyses showed that HSP90 positive expression levels were significantly higher in gastric cancer patients with increased tumor size (*P* = 0.001), tumor site (*P*<0.001), depth invasion (*P*<0.001), presence of lymph node metastasis (*P*<0.001) and stage of disease (*P*<0.001) (Table 1). No significant association was observed between gender, age, and grade of differentiation with HSP90 expression.

### Impact of HSP90 Overexpression on Invasion and Metastasis in Gastric Cancer

Notably, the correlation of prominent serosal invasion and lymph node metastasis with HSP90 positivity suggested a potential role of HSP90 in increased invasion and metastasis of gastric cancer. Therefore, we investigated the relationship of HSP90 and MMP-9 protein expression in gastric cancer.

**Figure 2:**
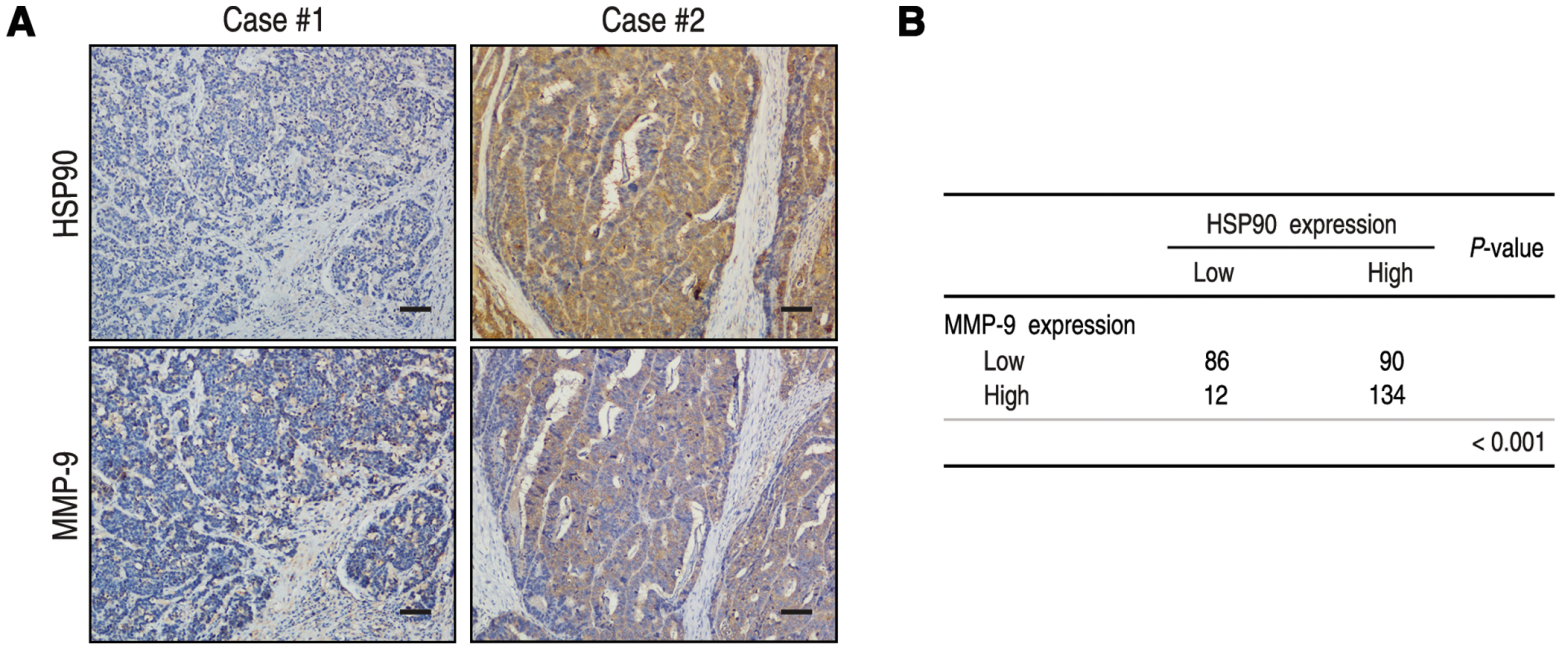
HSP90 and MMP-9 protein levels correlated in 322 advanced gastric cancer tissues. (**A**, **B**) IHC staining for HSP90 and MMP-9 was performed in tumors from 322 patients with advanced gastric carcinoma. Representative examples of HSP90 and MMP-9 staining in serial sections from the same tumor samples are shown in (**A**), and a summary of the results is shown in (**B**). The scale bar represents 200 µm.

The positive rates of HSP90 expression were 89.9% and 92.3% in the more prominent serosal invasion group (T3/T4) and more frequent lymph node involvement group (N1-3), while there were only 44.4% and 19.0% in T2 and N0 (*P*<0.001 and *P*<0.001, respectively) (Table 1). The level of HSP90 in T3 showed no difference with those in T4, and meanwhile, the expression of HSP90 had no significant differences among N1, N2 and N3 (datas not shown). In addition, HSP90 protein expression was significantly associated with MMP-9 expression in 322 gastric carcinoma tissues. Of 98 patients with low HSP90 expression, 86 patients (87.8%) had low MMP-9 expression, while 134 of 224 patients (59.8%) with high HSP90 expression also had high MMP-9 expression (*P*<0.001) (Fig. 2).

### Effect of Tumor HSP90 Protein Level on Prognosis

**Figure 3:**
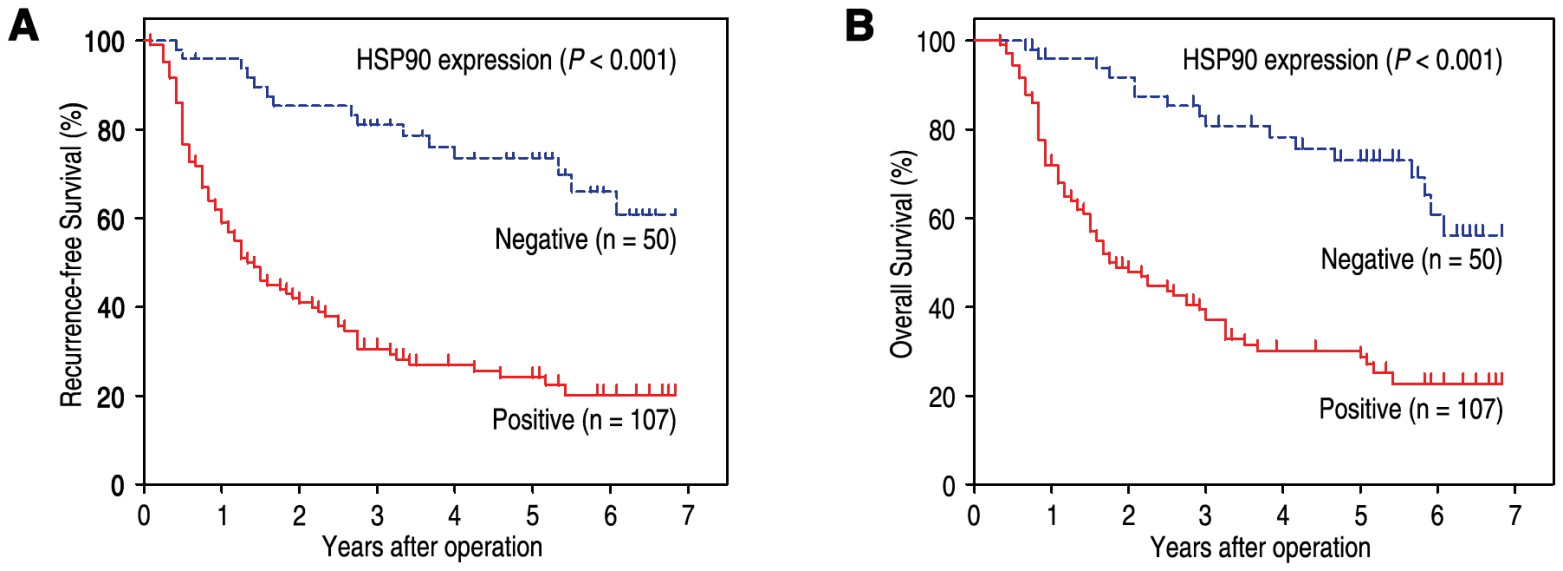
HSP90 overexpression indicates poor prognosis in advanced gastric cancer patients. RFS (**A**) and OS (**B**) curves were generated based on the HSP90 protein expression statuses in 157 gastric carcinoma samples.

Survival analysis showed that RFS and OS were significant different among 157 patients according to the expression of HSP90 (*P*<0.001 and *P*<0.001, respectively) (Fig. 3). The postoperative median RFS and OS were 27.0 months and 33.0 months, respectively. The postoperative median RFS and OS of patients with positive staining of HSP90 were 15.0 months and 20.0 months, while those of patients with negative staining of HSP90 were 60.5 months and 64.0 months. The 3-year and 5-year cumulative survival rates of patients with HSP90 negative expression were 83.1% and 75.7%, compared with 39.4% and 30.1% of patients with HSP90 positive expression, respectively (Table 2).

**Table 2:** Predictive Variables for Recurrence-Free Survival and Overall Survival of 157 Patients with Gastric Cancer.

		RFS rate (%)			OS rate (%)		
Variables	Number	3 y	5 y	*P*-value	3 y	5 y	*P*-value
Gender							
Male	96	47.2	41.8	0.939	52.6	44.7	0.830
Female	61	46.2	37.6		54.6	42.4	
Age (years)							
≤60	104	48.4	43.2	0.853	52.4	46.1	0.707
>60	53	43.8	34.7		55.5	40.3	
Size (cm)							
≤5.0	107	55.4	47.8	**0.002**	59.3	50.8	**0.002**
>5.0	50	28.8	24.4		40.8	29.2	
Tumor site							
Upper	35	33.8	27.1	0.064	46.2	32.6	0.090
Middle/Lower	122	50.4	43.8		55.4	47.0	
Differentiation							
Well/Moderate	57	44.2	39.8	0.839	49.5	43.8	0.598
Poor	100	48.3	40.1		55.6	43.8	
Depth of invasion							
T2	70	72.9	67.3	**<0.001**	72.6	68.5	**<0.001**
T3/T4	87	25.5	18.5		37.9	24.3	
Lymph node metastasis							
Negative	50	82.9	77.7	**<0.001**	84.8	79.4	**<0.001**
Positive	107	29.8	23.1		38.6	27.7	
HSP90 protein expression							
Negative	50	81.1	73.5	**<0.001**	83.1	75.7	**<0.001**
Positive	107	30.5	24.1		39.4	30.1	

To examine the impact of HSP90 overexpression on the RFS and OS, we performed univariate analysis of traditional clinicopathologic variables for prognosis. The results of univariate analysis were shown that significant variables in the RFS and OS analysis included HSP90 overexpression (*P*<0.001 and *P*<0.001, respectively), larger tumor size (*P* = 0.002 and *P* = 0.002, respectively), prominent serosal invasion (*P*<0.001 and *P*<0.001, respectively) and lymph node metastasis (*P*<0.001 and *P*<0.001, respectively) were positive prognostic factors for RFS and OS in gastric cancer patients (Table 2). However, gender, age, tumor site or differentiation status had no prognosis value on RFS and OS of patients with gastric cancer.

Furthermore, to evaluate the independent impact of HSP90 overexpression on RFS and OS, a multivariate Cox proportional hazards model was adjusted for tumor size, depth of invasion, lymph node metastasis and HSP90 expression. Our results demonstrated that HSP90 expression was an independent prognostic factor for both RFS (HR = 2.158, 95% CI: 1.165–3.999; *P* = 0.015) and OS (HR = 1.888, 95% CI: 1.022–3.486; *P* = 0.042) of patients with gastric cancer. Tumor size, depth of invasion and lymph node metastasis all had independent prognostic value in the multivariate analysis (Table 3).

**Table 3:** Multivariate Cox Regression Analysis for Recurrence-Free Survival and Overall Survival in Patients with Gastric Cancer.

	RFS (n = 157)		OS (n = 157)	
Variable	Hazard Ratios (95% CI)	*P*-value^a^	Hazard ratios (95% CI)	*P*-value^a^
Tumor size	1.578 (1.035∼2.406)	**0.034**	1.587 (1.034∼2.435)	**0.034**
Depth of invasion	2.007 (1.158∼3.479)	**0.013**	1.917 (1.096∼3.354)	**0.023**
Lymph node metastasis	3.379 (1.683∼6.785)	**0.001**	3.209 (1.594∼6.462)	**0.001**
HSP90 expression	2.158 (1.165∼3.999)	**0.015**	1.888 (1.022∼3.486)	**0.042**

Abbreviations: HSP90, heat shock protein 90; CI, confidence interval.

a Cox proportional hazard model regression. Bold values are statistically significant (*P*<0.05).

### Response of HSP90 in Neoadjuvant Chemotherapy

The difference in survival observed in patients with gastric cancer prompted us to question whether HSP90 expression was related to administration of chemotherapy. We detected HSP90 expression in diagnostic biopsy materials and matched surgical samples from 54 patients with gastric cancer.

**Figure 4:**
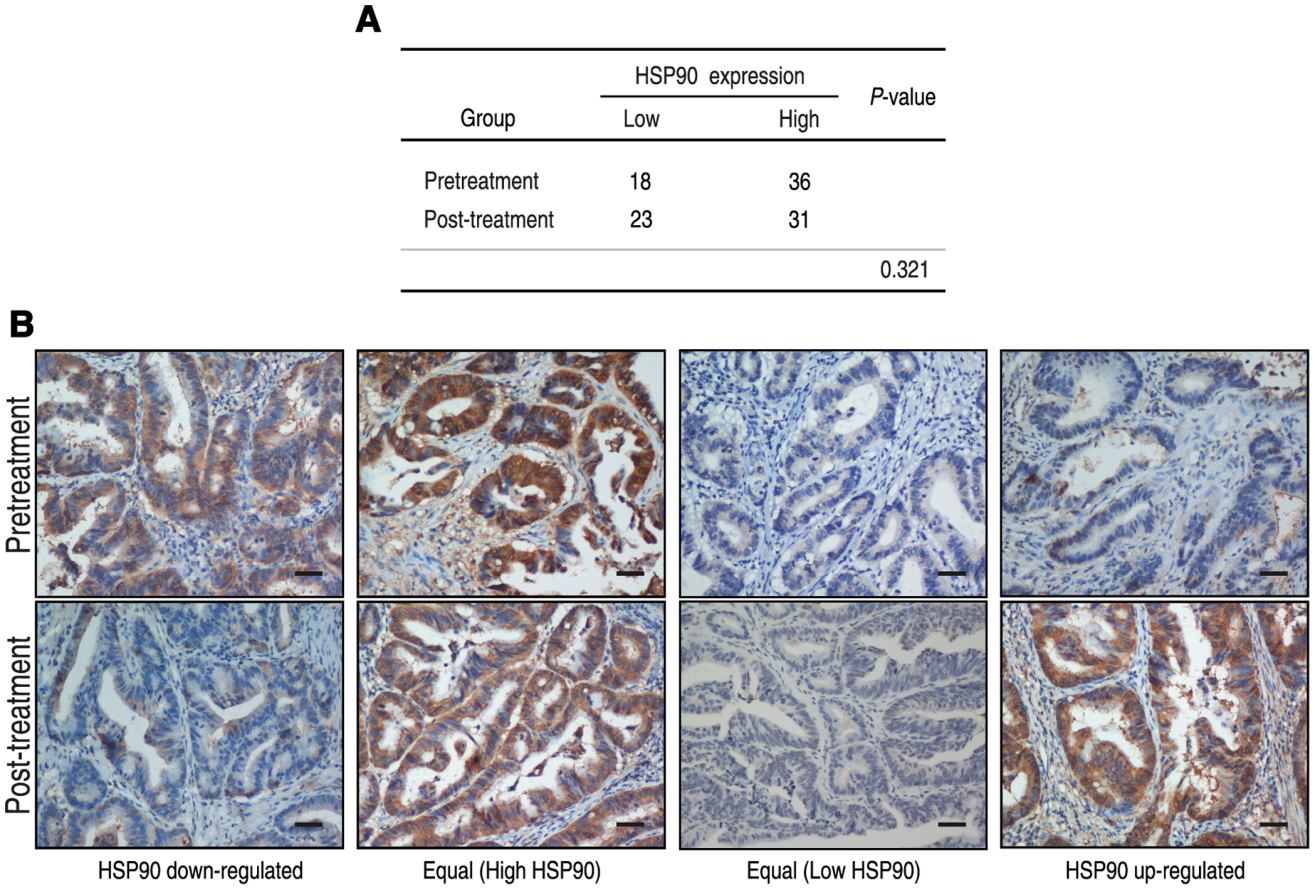
HSP90 responses to neoadjuvant chemotherapy. (**A**) The expression of HSP90 did not alter between the biopsy and tumor specimens (*P* = 0.321). (**B**) HSP90 staining in diagnostic biopsy material compared with matched tumor cores. The scale bar represents 100 µm.

HSP90 was expressed in 66.7% (36/54) of biopsies and 57.4% (31/54) of resection specimens. Neoadjuvant chemotherapy did not alter HSP90 expression between the biopsy and tumor specimens (*P* = 0.321) (Fig. 4). In 24 (44.4%) cases, HSP90 expression was identical in the biopsy and the matched specimens. 13 (24.1%) cases showed upregulation of HSP90 expression and 17 (31.5%) cases showed downregulation of HSP90 expression in the surgical samples compared with the biopsy material.

## Discussion

Heat shock proteins (HSPs) are found to be increased in many solid tumors and haematological malignancies. Their expression may play an important role in maintaining protein homoeostasis of malignant cells even in the hostile hypoxic and acidotic microenvironment of the tumor. Meanwhile, HSPs allow tumor cells to tolerate genetic alterations which would otherwise be fatal [16]. It has been reported that overexpression of HSPs is associated with poor prognosis, such as HSP27 in rectal cancer [17], HSP70 in pancreatic adenocarcinoma [18] and HSP90 in gastrointestinal stromal tumors [19]. Nevertheless, the role of HSP90 in gastric cancer has not been thoroughly elucidated.

In the present study, we described the prognostic value of HSP90 protein expression in advanced gastric cancer, and determined the relationship of HSP90 expression with invasion, metastasis, and chemotherapeutic response of gastric cancer patients. Immunohistochemistry was used to analyze the correlation of HSP90 protein expression with clinical pathological factors and prognostic effect of 322 gastric cancer patients. The result demonstrated that HSP90 protein levels were significantly associated with tumor size, tumor site, depth invasion, lymph node metastasis and clinical stages. In addition, Kaplan-Meier analysis showed that in general, HSP90-positive patients had worse prognosis than did HSP90-negative patients by both RFS and OS. Moreover, the multivariate Cox model analysis indicated that HSP90 expression status was identified as an independent risk factor for both RFS and OS. Our data suggested that HSP90 might play an important role in tumor prognosis and that HSP90 could be a potential prognostic factor of gastric cancer.

It has been known that degradation of extracellular matrix (ECM) was a signal for the beginning of invasion and metastasis, and MMPs are important molecules involved in ECM degradation during invasion and metastasis [20]. Chu et al. [21] reported that cancer MMP-9 was significantly correlated with depth of invasion and lymph node metastasis and that MMP-9-positive gastric cancer patients had worse outcomes than those with MMP-9-negative tumors. Moreover, it has been reported that HSP90 and MMP-9 can constitute as a complex in anaplastic large cell lymphomas, and MMP-9 could be activated by HSP90 to promote cell invasion. [22] Our results demonstrated that the expression of HSP90 and MMP-9 were correlated with each other, implying higher invasive and metastasizing activity in HSP90 high-expression cancer cells. HSP90 may be a key molecule for progression in gastric cancer. In addition, HSP90 was highly expressed in depth of invasion, especially in T3 and T4 carcinomas. As far as lymph node status was concerned, the patients with lymph node metastasis tend to show elevated HSP90 expression, while there was no statistically difference among N1, N2 and N3. Collectively, HSP90 expression in gastric cancer promotes tumor aggressiveness suggests that HSP90 might be a feasible target in cancer therapy.

Furthermore, we investigated the response of HSP90 in neoadjuvant chemotherapy, and found that neoadjuvant chemotherapy did not alter HSP90 expression between the biopsy and tumor specimens of gastric cancer. It suggested us that the HSP90 levels remain largely unchanged in chemotherapeutic effect, which afforded us the opportunity to make an important observation that the expression of HSP90 levels in the tumors was stable, no matter whether it received neoadjuvant chemotherapy or not. It has been reported that HSP90 confers resistance to chemotherapy in cancer [23]–[24]. Moreover, inhibition of HSP90 expression could reduce angiogenesis and gastric cancer cell proliferation, and overcome the resistance to chemotherapy [12]; [25]. Taken together, targeting HSP90 with chemical inhibitors could be considered in the treatment of gastric cancer, especially when HSP90 was highly expressed in gastric cancer.

In summary, we demonstrate overexpression of HSP90 may play an important role in tumor invasion, metastasis and prognosis, and might work as a promising target for prognostic prediction in gastric cancer.
